# Efficacy and safety of pulsed radiofrequency modulation of thoracic dorsal root ganglion or intercostal nerve on postherpetic neuralgia in aged patients: a retrospective study

**DOI:** 10.1186/s12883-021-02286-6

**Published:** 2021-06-24

**Authors:** Xuehua Huang, Yanfeng Ma, Weimin Wang, Yunxiu Guo, Bo Xu, Ke Ma

**Affiliations:** 1 Department of Pain, Xinhua Hospital, Shanghai Jiaotong University School of Medicine, Shanghai, PR China; 2 Department of Pain, The Seventh People’s Hospital, Shanghai University of Traditional Chinese Medicine, Shanghai, PR China; 3 Department of Pain, The Fourth People’s Hospital, Yancheng, Jiangsu PR China; 4 Department of Anesthesiology, Xinhua Hospital Chongming Branch, Shanghai, PR China; 5 Department of Anesthesiology and Surgical Intensive Care Unit, Xinhua Hospital, Shanghai Jiaotong University School of Medicine, Shanghai, PR China

**Keywords:** Pulsed radiofrequencies, Postherpetic neuralgia, Dorsal root ganglion, Intercostal nerve, The aged patients

## Abstract

**Background:**

Postherpetic neuralgia (PHN) is common in elderly patients and can be alleviated by pulsed radiofrequency (PRF). However, PRF treatments display different efficacy on different nerves. The purpose of this study was to evaluate the efficacy and safety of ultrasound-guided PRF modulation on thoracic dorsal root ganglion (DRG) or intercostal nerve (ICN) for PHN in aged patients and to provide a theoretical basis for clinical treatment.

**Methods:**

We classified aged patients into two groups, DRG group and ICN group, based on the needle tip position. Visual analogue scale (VAS) and concise health status questionnaire (Short-form 36 health/survey questionnaire, SF-36) were used to evaluate the pain intensity and the life quality of the patients before and 2, 4 and 12 weeks after the PRF treatments. We also recorded the adverse reactions during the treatments.

**Results:**

After the PRF treatment, the scores of VAS and SF-36 (assessing general health perception, social function, emotional role, mental health, and pain) improved significantly in both groups (*P* < 0.05). The mean VAS score in the DRG group was significantly lower than that in the ICN group 2 weeks after treatment, and remained for 12 weeks. The SF-36 scores in the DRG group were significantly higher than those in the ICN group (*P* < 0.05). We found a similar incidence of adverse reactions between the two groups (*P* > 0.05).

**Conclusions:**

PRF therapy is safe and effective for elderly patients with postherpetic neuralgia. However, PRF treatment in dorsal root ganglion is superior to that in intercostal nerve with improving VAS and SF-36 scores to a greater extent in older patients.

**Trial registration:**

ChiCTR2100044176.

## Introduction

Postherpetic neuralgia (PHN) is a chronic pain caused by varicella-zoster virus infection, which always occurs among older adults. People suffer from PHN over 50 years old accounts for 12.5% patients with PHN [1]. And PHN prevalence increases with advancing age [1, 2].

The typical clinical symptoms of PHN is a persistent sharp or burning pain with spontaneous pain [3], which seriously affects the elderly patients’ quality of life. Its pathogenesis is complicated making it difficult to be treated [4]. A variety of treatments is applied into the clinical practice, which include drug therapy and minimally invasive therapeutic procedures (nerve blocks, pulsed radiofrequency, neurolysis, and so on). Drug treatments are unlikely to fundamentally solve the patients’ clinical symptoms, and some patients have to cease the medications due to their side effects, especially the elderly [5].

Pulsed radiofrequency (PRF) is a minimally invasive technique that applies pulsed current (300–500 kHz) to the target nerve. The current is delivered in a pulse of 20 ms (45 V’ voltage) followed by a silent period of 480 ms to avoid heat lesions [6]. Recent studies have confirmed the beneficial effects of PRF against post-operative pain, peripheral neuropathic pain, and postherpetic neuralgia [7–10]. The thoracic nerves (T1-12) are the most commonly affected by PHN with an incidence of up to 50% cases [5]. Studies have shown that both DRG and ICN with PRF treatments are effective in the treatment of thoracic postherpetic neuralgia [8]. However, no studies have compared the analgesic effects of these two methods in aged patients. The purpose of this study was to compare the clinical efficacies and security of these two PRF therapy methods for elderly patients with PHN.

## Methods

### Patients

The Ethics Review Committee of the Xinhua Hospital affiliated to the Medical College of Shanghai (Jiaotong University) approved this retrospective analysis (XHEC-D-2020–166). The need for a written consent from patients was waived because we ensured all the information and treatment records of the aged patients were kept anonymous to all researchers involved. The United Nations has agreed that 65 + years may be usually denoted as old age in developed country and 60 + as old age in developing country [11]. As a developing country, we selected 60 + patients as the object of the study. We collected clinical data from the hospital database and analyzed the records of all PHN patients older than 60 years who received thoracic PRF treatment in the pain department between June 2017 and June 2020.

The inclusion criteria were the following: (1) pain duration > 1 month; (2) thoracic herpes zoster infection; (3) age > 60 years; and (4) VAS score > 4 after conservative treatment (including oral medication).

The exclusion criteria were: (1) history of cancer; (2) systemic immune disease; (3) incomplete 3-month follow-up data; (4) receiving epidural catheter therapy or spinal cord electrical stimulation within 3 months after the PRF treatments; (4) receiving two or more times of PRF treatments.

### PRF procedure

The patients were putted in the prone position on the operating table with a comfortable pillow under their chest. The PFR was carried out under large-scale Digital Subtraction Angiography (DSA, PHILIPS Company, SN:60536M151838, Holland) and B-scan ultrasound (Sonosite Company, Sonosite Edge, America) imaging. We used a PM-230 pain management generator (Baylis Medical Company, Montreal, Canada) and a 21-gauge, straight, sharp PRF cannula needle with a 5-mm exposed tip.

**Fig. 1:**
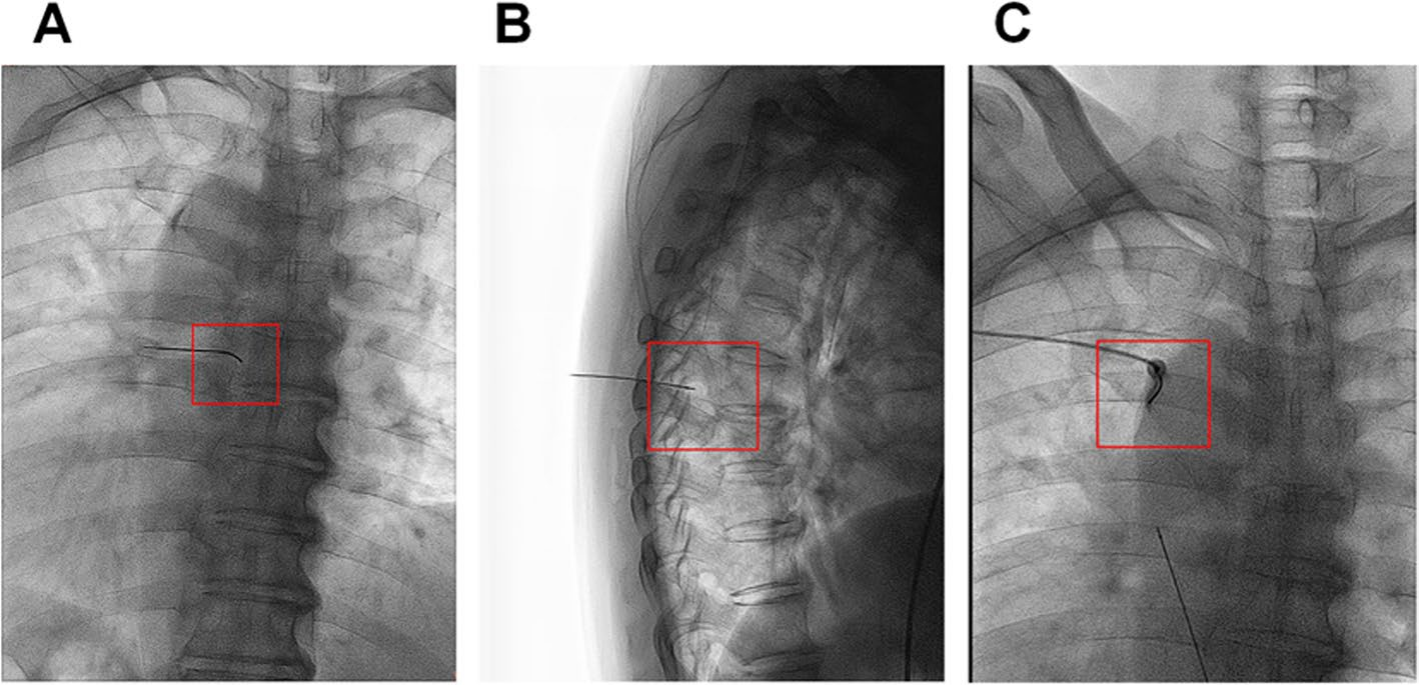
X-ray photo of PRF treatment. **A** X-ray imaging of PRF treatment on DRG in the anteroposterior view. **B** X-ray imaging of PRF treatment on DRG in the lateral view. **C** X-ray imaging of PRF treatment on ICN in the anteroposterior view. PRF, pulsed radiofrequency. DRG, dorsal root ganglion. ICN, intercostal nerve radiofrequency

For the DRG treatment group: The puncture needle entered into the thoracic paraspinal space under the guidance of B-scan ultrasound (Huasheng portable ultrasound instrument, Shenzhen, China). We further adjusted the needle tip based on the DSA scanning images. Once needle tip right below the lateral edge of the vertebral pedicle in the anteroposterior view (Fig. 1A) and on the upper quadrant of the dorsal side of the intervertebral foramen in the lateral view (Fig. 1B), the position of the needle tip was controlled by sensory and motor nerve stimulation before further operation.

For the ICN group, the puncture needle entered into the low edge of the angulus costae of the corresponding intercostal nerve (Fig. 1C). After confirming the position using sensory and motor nerve electrical stimulation, the patients received PRF treatment for 3 cycles. The working mode of PRF was: 15–20 ms pulse electric current, 45 V’ voltage at 42 °C for 120 s. Impedance was maintained at less than 500 Ω throughout the procedure. The vital signs of the patients (blood pressure, respiration, pulse, body temperature, and consciousness) were closely monitored during and after operation.

### Data collection

We collected and analyzed the demographic data including age, gender, presence of comorbidities (such as hypertension and diabetes mellitus), and the duration and degree of pain. We also recorded and compared the VAS, SF-36 scores and side effects at 2, 4 and 12 weeks after treatment between the two groups. SF-36 scores were used for evaluating the life quality, higher scores indicated better quality of life [12], including general health perception, social function, emotional role, mental health index, pain index, physical function, physical role, and vitality. We also recorded data on adverse reactions of the treatments.

### Statistical analysis

SPSS 26.0 software was used to analyze all the data in this study. Continuous variables with normal distribution were expressed as Mean ± SD, while non-normally distributed data were expressed as median ± interquartile range. We chose repeated measurement variance analysis to compare continuous measurement of data between the two groups. Chi-square tests were used to compare composition ratios of two different groups. *P*-values < 0.050 is regarded as statistically significant.

## Results

**Fig. 2:**
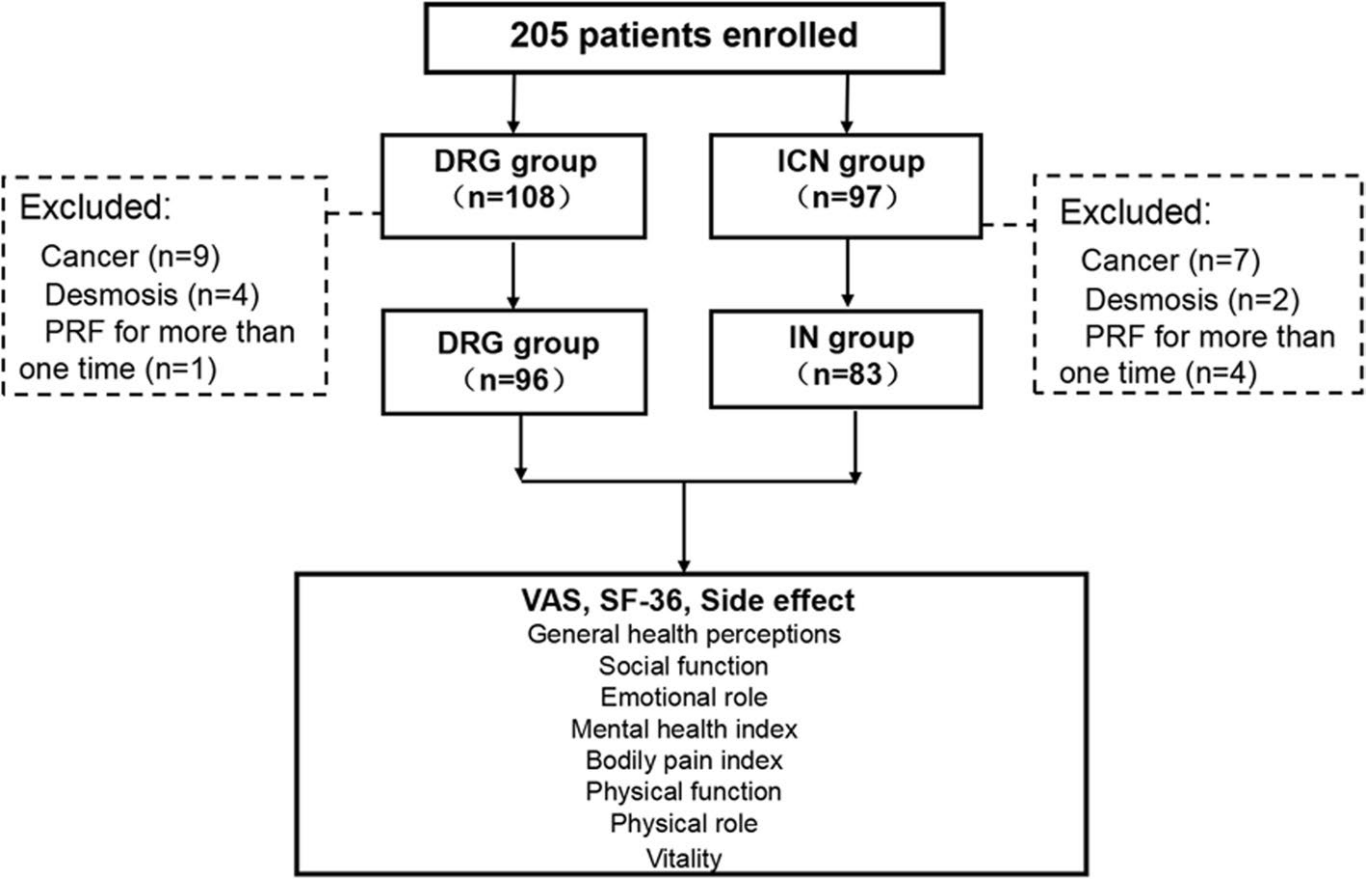
Flow diagram of study patient inclusion. PRF, pulsed radiofrequency. DRG, dorsal root ganglion. ICN, intercostal nerve

In this retrospective study, we collected the data of 205 aged patients who underwent PRF. Among the 108 patients who underwent DRG PRF treatments, 12 patients were excluded due to 9 patients with malignant tumors, 2 patients with systemic immune disease, and 1 patient with two times of PRF treatments. At last, we included 96 patients. For the 97 aged patients who underwent ICN PRF, 7 patients had malignant tumors, 3 patients had desmosis, and 4 patients had two times of PRF treatments. In the end, we analyzed data from 83 patients (Fig. 2).

Table 1 described the demographic characteristics of the aged patients in the study. We found no significant differences in age, gender, pain duration, or other medical history features between the patients in the DRG and ICN groups (*P* > 0.05). The differences in VAS and SF-36 scores between the two groups before treatments were not statistically significant (*P* > 0.05).

**Table 1:** Clinical characteristics of the study participants

**Characteristic**	**DRG PRF group (*n* = 96)**	**IN PRF group (*n* = 83)**	***P***
Age, mean (SD), year	70.0 (7.5)	70.7 (7.3)	0.373
Sex ratio (F:M)	48:48	45:38	1.000
**Coexisting conditions, no**			
Hypertension	21	26	0.207
Diabetes	16	13	0.983
Pain duration (months), (median, interquartile range)	2 (1–5)	2 (1–5.75)	0.283
VAS (mean, SD)	6.0 (1.26)	6.02 (1.29)	0.454
SF-36 (mean, SD)			
General health perception	43.06 (6.52)	43.45 (6.62)	0.692
Social function	41.20 (6.34)	41.34 (6.49)	0.884
Emotional role	44.92 (6.43)	45.11 (6.48)	0.844
Mental health index	41.35 (5.44)	41.01 (5.61)	0.682
Bodily pain index	35.02 (6.62)	35.43 (6.44)	0.676
Physical function	54.41 (6.27)	54.16 (6.33)	0.791
Physical role	55.01 (6.07)	54.94 (6.30)	0.940
Vitality	41.61 (6.64)	41.94 (6.43)	0.737

**Fig. 3:**
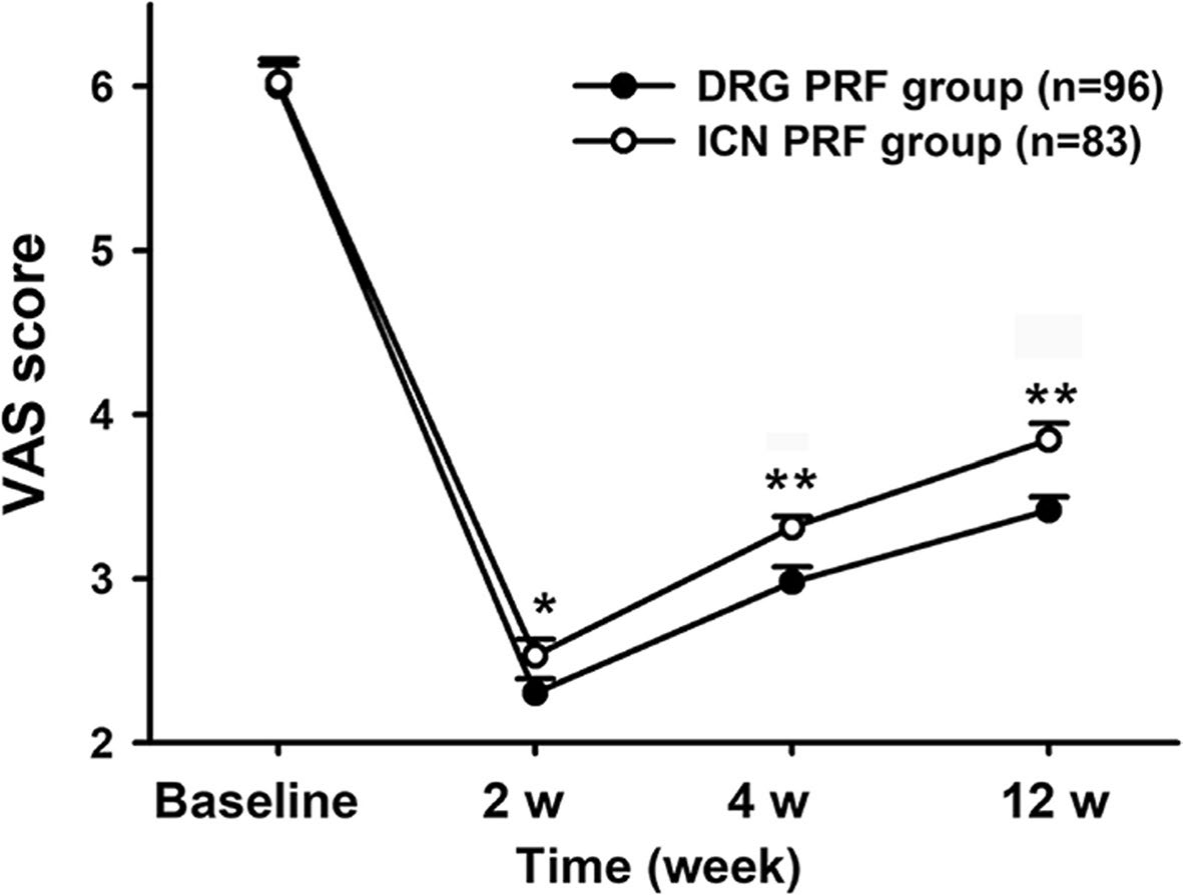
The changes in VAS of the two groups. VAS, visual analogue scale DRG group, dorsal root ganglion pulsed radiofrequency group, ICN group, Intercostal nerves radiofrequency group. *P < 0.050 compared to DRG group, **P < 0.010 compared to DRG group. PRF, pulsed radiofrequency. DRG, dorsal root ganglion. ICN, intercostal nerve. VAS, Visual Analogue Scale

After treatments for 2 weeks, VAS scores in the DRG group were significantly lower than those in the ICN group, and the score gap increased at 4 weeks and remained so until 12 weeks after treatments (*P* < 0.01; Fig. 3).

**Fig. 4:**
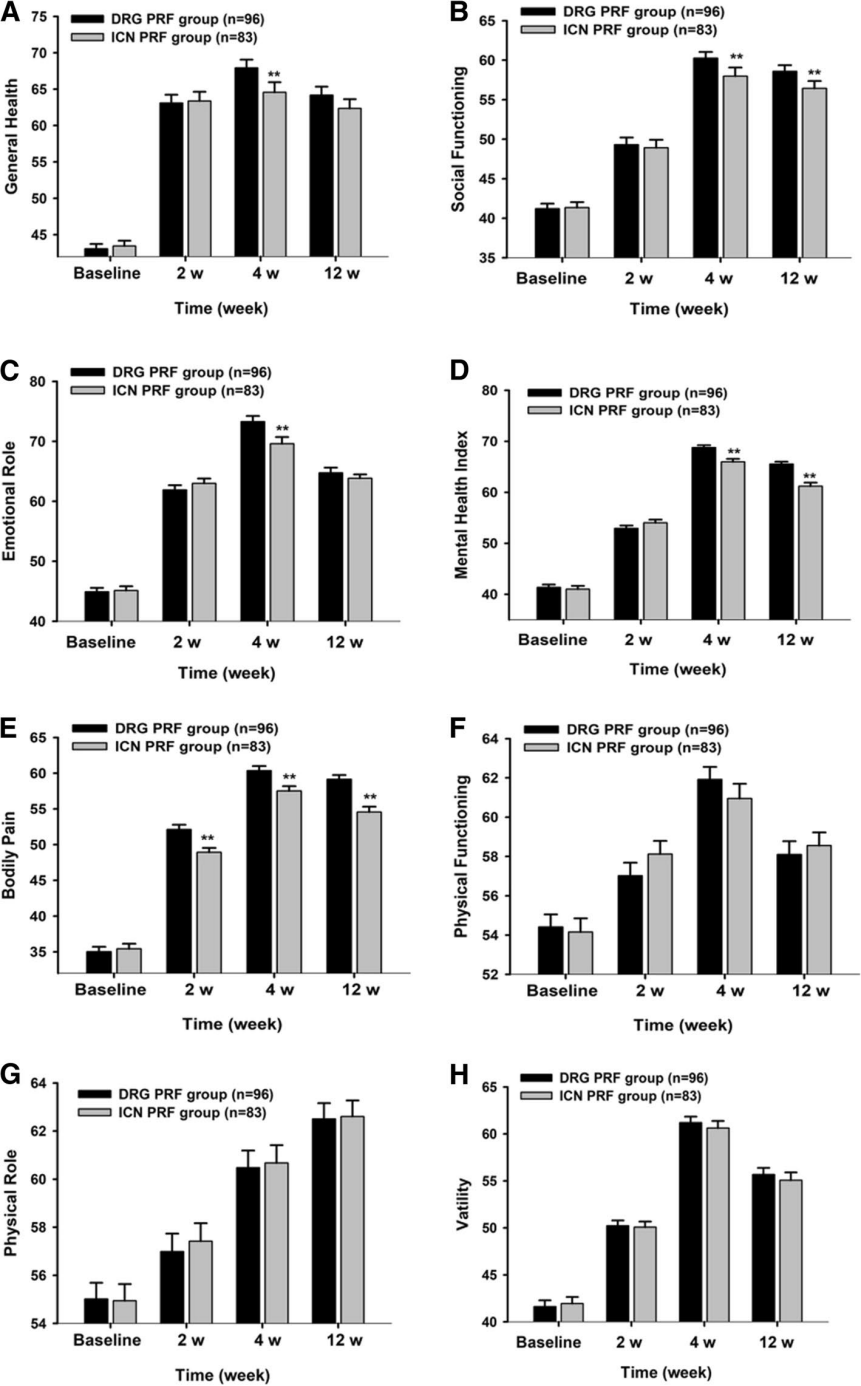
The changes in SF-36 of the two groups. DRG group, dorsal root ganglion pulsed radiofrequency group. ICN group, intercostal nerve pulsed radiofrequency group group. **P < 0.010 compared to DRG group. SF-36, Short-form 36 health/survey questionnaire

We compared SF-36 scores between two groups at 2, 4 and 12 weeks after treatments and found that pain index in the DRG treatment group was significantly lower than that in the ICN treatment group at 2 weeks after treatments and remained so until the 12 weeks post-treatment (*P* < 0.01). The levels of general health perception, social function, emotional role, and mental health index in the DRG treatment group were generally better than those in the ICN treatment group (*P* < 0.05). The scores for physical function, physical role, and vitality were similar in both groups after treatment (*P* > 0.05; Fig. 4).

During the operation, one patient (1%) developed a pneumothorax in the ICN group. And one patient (1%) was found with the needle penetration of the subarachnoid space in the DRG group. The patient with pneumothorax completely recovered with oxygen therapy for 2 days. The patient where the needle penetrated the subarachnoid space was kept supine for 6 h and had no further discomfort. Considering that we used B-scan ultrasound and DSA during the whole operation, the needle tip position was carefully controlled, and complications are very rare and mild. No other adverse reactions occurred, such as spinal cord injury, hematoma, limb dyskinesia and so on.

## Discussion

Our study results indicate that PRF treatments on different targets have different effects on PHN in aged patents. In PHN, PRF treatments at DRG seems to be superior to that at ICN, as seen by the significant VAS and SF-36 score gap between the two groups.

PHN is a kind of pain that persists 1 month after an acute herpes zoster event, and the pain in some patients may last more than 10 years [1, 2]. Varicella-zoster virus, which causes herpes zoster, persists in the spinal nerve DRG of patients for a long time after infecting the human body. Latent viruses duplicate quickly when the body’s immunity becomes lower with age [1]. Therefore, older people are more likely to develop herpes zoster and its complications PHN. Clinical studies have found that the incidence of PHN is as high as 60 to 75% [2] in patients over 60 years and that it seriously impacts their physical and mental health and their quality of life [13, 14]. Hence, the study focused on old patients with PHN to choose the better therapy method to improve their quality of life. The DRG suffers the most damage during PHN [15]. Firstly, the reactivated varicella-zoster virus in the DRG proliferates and destroys axons, causing demyelination and ion channel dysfunction. The damaged sensory nerves can generate abnormal electrical impulses that are transmitted to the spinal cord transmitting pain and pain hypersensitivity [16, 17]. There are lots of inflammatory cells invading into DRG of patients with PHN [18]. Then, the inflammatory mediators transmit pain information and causes central sensitization.

The mechanisms of PRF on pain relief are complicated. Previous studies have indicated that the analgesic effect of PRF is due to the pulsed current and the biological effects. It has been demonstrated that PRF applied to the rat cervical DRG increased c-Fos immunoreactivity in the laminae of the spinal dorsal horn [19], which indicated nerve fibers have been activated by high electric fields. Hamann et al., also has comfirmed the biological effects of PRF, which could upregulated of ATF-3 (activating transcription factor-3, a marker of cellular stress) in the DRG neuronal bodies after applied to the L4 DRG compared to sham-operated DRG [20]. Recent studies have demonstrated that PRF could enhance the descending noradrenergic and serotonergic inhibitory pathways [21], which are involved in the modulation of neuropathic pain. From the available evidence, PRF appears to be temperature independent to regulate biological effects of cell morphology, synaptic transmission, and pain signaling.

Based on these effects, PRF has been widely applied for the treatment of PHN. Ding et al. [22] have indicated that PRF of the thoracic DRG under CT guidance is safe and effective for various PHN treatments. Ma et al. [23] have verified that PRF through the angulus costae to intercostal nerves is an effective approach for thoracic PHN and that it can last for approximately half a year. Similar with previous study, this study also confirms that PRF at DRG or ICN is effective against thoracic PHN. Different with previous study, we mainly focus on the older patients who are more susceptible to PHN and suffer longer pain than younger persons. The aged also have important pharmacokinetic and pharmacodynamic changes [24]. Renal function always decreases with aging, and drugs metabolized by the kidney should be adjusted dosage in these patients with renal impairment [25]. The aged patients always have an increased sensitivity to drugs acting on the CNS, with a result of increasing central side effects of some drugs [26]. Hence, the old patients always tolerate the medication less well than young patients. Secondly, we compare the efficacy of PRF at DRG or ICN to thoracic PRF and find PRF at DRG with better analgesic effect and higher life quality in the aged patients. In addition, both B-scan ultrasound and DSA are used during the operation, the location could be more accurate and the complications could be less.

Indeed, PRF at ICN can also relieve PHN. When the body’s immunity reduces, the latent virus always replicates along the sensory nerve reaching the corresponding skin area, which results in peripheral nerve necrosis, inflammation, and demyelination [27]. Peripheral nerve sensitization plays a particularly important role in neuropathic pain. PRF reversibly blocks the transmission of nerve impulses in small or unmyelinated nerve fibers in peripheral nerve [28, 29]. Animal studies have shown that PRF causes obvious changes in the axons of C fibers by inducing mitochondria edema, abnormal ATP metabolism, changes of ion channel [30–32]. As a result, PRF at ICN decreases VAS and SF-36 scores in the old patients of our study. PRF was reported to have no damage to the axonal adventitia [33]. It is safe to use PRF of peripheral nerve to treat PHN. However, in our study, PRF of DRG is better than that of ICN.

The results of this study show that the scores of VAS and SF-36 in the DRG group were better than those in the ICN group. DRG neurons have the primary receptors of pain and temperature perceptions [5, 18]. The electrical signals of pain in the trunk are first integrated at DRGs and then transmitted to the spinal cord, which finally arrive in the central nervous system [17]. PRF can stimulate DRGs by intermittent pulse currents and block the pain signal transduction [22]. In addition, PRF could form a high voltage field around DRGs, which further inhibits the activation of glial cells [34]. However, the mechanism that PRF of DRG is better than that of ICN to treat PHN still needs further researches. Besides, as a retrospective study, it is difficult to avoid selectivity bias. Prospective studies are still needed to confirm the results of our research.

In this retrospective study, both B-scan ultrasound and DSA are used during the whole operation, complications are very rare in both groups. However, it’s worth noting that a patient developed pneumothorax with lung tissue compressed less than 30% [35]. After oxygen therapy (40% oxygen concentration) for 2 days, the patient was found that pneumothorax was absorbed by X-ray scan. Another patient, where the needle penetrated the subarachnoid space, was left supine for 6 h and monitored his vital signs for 2 days. He did not have any discomfort although some patients will develop headache due to cerebrospinal fluid leakage.

In summary, the analgesic effects of PRF treatments on postherpetic neuralgia are relative to their target positions. PRF treatments in dorsal root ganglion is superior to that in intercostal nerve with improving VAS and SF-36 scores to a greater extent in older patients.

## Conclusion

PRF treatment in dorsal root ganglion is recommended for older patients with postherpetic neuralgia in thoracic segment.

## Data Availability

Data are available from the corresponding author on reasonable request.

## Abbreviations

PHN: Postherpetic neuralgia
DRG: Dorsal root ganglion
ICN: Intercostal nerve
VAS: Visual analogue scale
SF-36: Concise health status questionnaire
